# Upper Limit on the Thermodynamic Information Content of an Action Potential

**DOI:** 10.3389/fncom.2020.00037

**Published:** 2020-05-13

**Authors:** Sterling Street

**Affiliations:** Department of Biology, University of Georgia, Athens, GA, United States

**Keywords:** Landauer's principle, information, thermodynamics, action potential, neurons, entropy

## Abstract

In computational neuroscience, spiking neurons are often analyzed as computing devices that register bits of information, with each action potential carrying at most one bit of Shannon entropy. Here, I question this interpretation by using Landauer's principle to estimate an upper limit for the quantity of thermodynamic information that can be processed within a single action potential in a typical mammalian neuron. A straightforward calculation shows that an action potential in a typical mammalian cortical pyramidal cell can process up to approximately 3.4 · 10^11^ bits of thermodynamic information, or about 4.9 · 10^11^ bits of Shannon entropy. This result suggests that an action potential can, in principle, carry much more than a single bit of Shannon entropy.

## 1. Introduction

“*The fundamental constraint on brain design emerges from a law of physics. This law governs the costs of capturing, sending, and storing information. This law, embodied in a family of equations developed by Claude Shannon, applies equally to a telephone line and a neural cable, equally to a silicon circuit and a neural circuit.”* – P. Sterling and S. Laughlin (Sterling and Laughlin, 2015)

In many areas of computational neuroscience, neurons are often analyzed as binary electrochemical switches (DeWeese et al., 2003; Victor, 2006; Jensen et al., 2013; Mayfield, 2013; Sterling and Laughlin, 2015; Gupta and Bahmer, 2019). At this level of abstraction, a spiking neuron can be treated as a memory system with two stable positions. The neuron may be firing, in which case its state is typically labeled as a 1, or the neuron may be resting, in which case its state is typically labeled as a 0. Since the probability that a neuron will fire an action potential is influenced by many different unknown factors (such as the neuron's temperature, its firing threshold, its degree of connectivity with presynaptic inputs, and so forth), the distinction between a firing state and a resting state can be studied as a binary random variable in Shannon's theory of communication. Thus, it is often implicitly assumed that a single action potential carries a Shannon entropy of

(1) $$\begin{array}{l} H=p\;{\text{log}}_{2}\left(\frac{1}{p}\right)+\left(1-p\right)\;{\text{log}}_{2}\left(\frac{1}{1-p}\right) \end{array}$$

where *H* is the number of bits of Shannon entropy in an action potential, and *p* is the action potential's initial probability. At a maximum, *H* = 1 bit.

Numerous hypotheses, models, and theories use the above equation for the Shannon entropy of a binary random variable as a starting point for more sophisticated analyses of neuronal information content (Borst and Theunissen, 1999; Arcas et al., 2003; Victor, 2006; Sharpee and Bialek, 2007; Jensen et al., 2013; Jung et al., 2014; Sengupta et al., 2014). For example, the popular “direct method” for calculating the information content of a spike train begins by dividing its total duration into a number of evenly spaced time bins. The presence or absence of an action potential within each time bin is then represented as a 1 or 0 (Victor, 2006). In the equation above, spike probability then becomes the product of recorded firing rate *r* and time bin size Δ*t*, so that *p* = *r*Δ*t* (Rieke et al., 1999; Arcas et al., 2003; Sterling and Laughlin, 2015). Although the quantity of information carried by the spike train in this method depends on the chosen time bin size, this parameter is often chosen to allow each bin to carry at most one spike (Victor, 2006). Since this method studies the presence or absence of an action potential within each bin as a binary variable, each individual spike usually contributes on the order of one bit of information to the structure of a spike train.

A second method for studying the information in neuronal signals, which is also based on Shannon's measure of entropy, is to quantify the mutual information in the correlation between a neuron's stimulus probability and its response probability (London et al., 2002; Victor, 2006; Jensen et al., 2013; Pregowska et al., 2015; Azarfar et al., 2018). Many variations of this method exist, and there are numerous ways to measure the degree of correlation between neuronal stimulus and response. Nevertheless, as standard measures of mutual information are maximized at one bit of Shannon entropy (Timme and Lapish, 2018), this method also generally carries the implicit assumption that each individual action potential within a spike train carries on the order of one bit of information about the stimulus with which it is correlated. Many other methods beyond the two described here can be used to quantify the information content of action potentials and spike trains. These include methods based on measures such as informational complexity, information transmission rate, Bayesian information, transfer entropy, and maximum entropy production (Victor, 2006; Chen, 2013; Li and Li, 2013; Crutchfield et al., 2015; Timme and Lapish, 2018). Many, though certainly not all, of these analyses are subject to the same essential criticism, as they often involve characterizing a spike train as a sequence of binary random variables.

This common assumption that an action potential can carry at most one bit of Shannon entropy about the structure of a spike train by virtue of being a binary random variable is certainly useful in many contexts. For this reason, the goal of this paper is not to argue that this assumption is necessarily erroneous or unjustified. Rather, I hope to explain why this interpretation of the action potential as a simple 1 or 0 may be misleading, at least from the perspective of fundamental physics. The next section discusses how the physical information content of an action potential is limited at a deeper level by the laws of physics.

## 2. What is Information?

“*It from bit. Otherwise put, every it — every particle, every field of force, even the spacetime continuum itself — derives its function, its meaning, its very existence entirely — even if in some contexts indirectly — from the apparatus-elicited answers to yes or no questions, binary choices, bits.”* – J. Wheeler (Wheeler, 1990)

As a prelude to the calculation that follows, it is worth taking a step back to elaborate on some of the physical meanings of the word *information*. According to an influential paradigm in contemporary theoretical physics, the bit of information is the ultimate irreducible building block of the physical world (Wheeler, 1990; Bekenstein, 2003; Brukner and Zeilinger, 2005; Chiribella et al., 2012; Lloyd, 2013; Davies and Gregersen, 2014; Rovelli, 2015; Vedral, 2018; Glattfelder, 2019; Smolin, 2020a). From this perspective, records of distinguishable events and interactions are even more fundamental than such entities as particles, fields, and forces. A bit of information, then, can be understood conceptually as any distinction able to be recorded about the outcome of an event with two mutually exclusive possibilities. The spin of an electron, the energy level of a two-level atom, and the linear polarization of a photon are all examples of bits of potential physical information (Schumacher and Westmoreland, 2010).

Given that an action potential is a collection of a large number of microscopic events involving systems such as electrons, ions, atoms, and molecules, it seems challenging to reconcile the claim that an action potential can hold at most one bit of information with modern information physics. While it may be convenient to study an action potential as if it were a simple binary random variable abstractly labeled as a 1 or 0, let us remember the words of Landauer (1996a). As he wrote, “Information is not a disembodied abstract entity; it is always tied to a physical representation. It is represented by engraving on a stone tablet, a spin, a charge, a hole in a punched card, a mark on paper, or some other equivalent. This ties the handling of information to all the possibilities and restrictions of our real physical word, its laws of physics and its storehouse of available parts.” In other words, when attempting to quantify information in physical systems, we must be mindful of the fact that bits of physical information correspond to real degrees of freedom. From a pancomputationalist perspective, the bits of thermodynamic information that are processed by an action potential can be identified as any physical distinctions that are acquired, changed, or lost by the neuron and its information-storing subsystems over the duration of the action potential.

Identifying any recorded distinctions in a spiking neuron as potential bits of information introduces the question of whether these bits evolve reversibly or irreversibly during an action potential signaling cycle. Although reversible (information-conserving) computation by neurons and their subsystems is theoretically possible, much evidence indicates that action potentials perform computations that are irreversible (information-erasing) with respect to the cell and its information-storing subsystems. As only one justification for this assertion, consider that information-processing systems which evolve according to fully reversible dynamics usually must be isolated from the environment and kept at low temperatures (Audretsch, 2008; Schumacher and Westmoreland, 2010; Schlosshauer, 2014). Neurons and their basic components, however, are warm, open, and noisy systems that interact heavily with their environments. A second way to arrive at the conclusion that action potentials are likely to process information irreversibly is to consider that reversible computation requires no energy expenditure (Landauer, 1996b; Bennett, 2003). Action potentials, however, require large amounts of energy (Attwell and Laughlin, 2001). As a third perspective, the brain is a dissipative structure (Kondepudi et al., 2017) in the sense that it maintains organization by transforming relative information into relative entropy through the process of erasure (Aur and Jog, 2007; Perez Velazquez et al., 2019).

If we hope to quantify the information content of an action potential, we must take these factors into account in order to be consistent with the physics of computation. Even though models that ignore the many microscopic degrees of freedom involved in the transmission of an action potential have led to great advances in our understanding of the brain, they may rely on physically problematic assumptions.

## 3. Landauer's Principle

“*Thermodynamic entropy and Shannon entropy are conceptually equivalent: the number of arrangements that are counted by Boltzmann entropy reflects the amount of Shannon information one would need to implement any particular arrangement. […] When the two entropies are calculated for the same degrees of freedom, they are equal.”* – J. Bekenstein (Bekenstein, 2003).

By illuminating surprising connections between information theory and thermodynamics, the information paradigm in physics has reinvigorated the study of heat. An excellent example of one such connection can be found in the similarity between equations at the foundation of each field. Consider Shannon's equation for the entropy contained within a set of discrete random variables (Shannon, 1948), which was originally developed to quantify the amount of surprisal in the outcome of one or more probabilistic events:

(2) $$\begin{array}{l} H=K\overset{N}{\underset{i=1}{\sum }}p_{i}\;log_{2}\left(\frac{1}{p_{\text{i}}}\right) \end{array}$$

where *H* is Shannon entropy, *K* is a positive constant, *p*_*i*_ is the probability of the *i^th^* possible outcome, and *N* is the number of mutually exclusive alternative outcomes.

It was only after a conversation with the physicist John von Neumann that Shannon chose to name this quantity “entropy,” after its resemblance to an equation introduced by Boltzmann and Gibbs nearly eight decades earlier (Petz, 2001). Long before the introduction of Shannon's information theory, the equation below had been developed as a way to quantify the amount of information that a macroscopic observer lacks about the microscopic configurations of a collection of particles:

(3) $$\begin{array}{l} S=k_{B}\overset{N}{\underset{i=1}{\sum }}p_{i}\;ln\left(\frac{1}{p_{\text{i}}}\right) \end{array}$$

where *S* is thermodynamic entropy, *k*_*B*_ is Boltzmann's constant, *p*_*i*_ is the probability of the *i^th^* microscopic configuration, *ln* is the natural logarithm, and *N* is the number of potential microscopic configurations that may underlie a macroscopic observation.

Ultimately, these and other overlapping themes between information theory and thermodynamics led Szilard, Landauer, Brillouin and others to what is now known as Landauer's principle, a form of information-energy equivalence that applies to any physical system processing information irreversibly (Bennett, 2003; Parrondo et al., 2015; Bormashenko, 2019a; Ribezzi-Crivellari and Ritort, 2019). This principle states that, when a memory system generates entropy by erasing information, the energy of the environment increases by at least:

(4) $$\begin{array}{l} \Delta E_{\text{env}}\geq k_{\text{B}}T\Delta S_{\text{sys}} \end{array}$$

where Δ*E*_env_ is energy dissipated into the environment, *k_B* is Boltzmann's constant, *T* is absolute temperature, and Δ*S*_sys_ is thermodynamic entropy generated (equivalent to the information that has been erased from the memory system). Here, *k*_*B*_ = 1.38064852 · 10^−23^ J/K. Since thermodynamic entropy is hidden information (Maruyama et al., 2009), Landauer's principle can also be written as

(5) $$\begin{array}{l} \Delta E_{\text{env}}\geq -k_{B}T\Delta I_{\text{sys}} \end{array}$$

where Δ*S*_*sys*_ = –Δ*I*_*sys*_, since a memory system's loss of thermodynamic information is equivalent to the generation of thermodynamic entropy. From these considerations, it can be shown that there exists a lower limit (Landauer, 1996b, 1999; Ribezzi-Crivellari and Ritort, 2019) on the amount of energy that is released when a physical system fully clears distinctions from its memory. In the case of a spiking neuron, for example, distinctions used to store information could include the configurations of proteins such as enzymes and ion channels, the locations of charged particles relative to a membrane, or the energy states of various molecules involved in action potential signaling. In any case, the quantity of energy released when a set of distinctions is erased is proportional to the quantity of thermodynamic information that they stored:

(6) $$\begin{array}{l} \Delta E_{\text{env}}\geq N\;k_{B}T\left[\;p\;ln\left(\frac{1}{p}\right)+\left(1-p\right)\;ln\left(\frac{1}{1-p}\right)\right] \end{array}$$

where *p* is the probability of the first of two mutually exclusive alternative physical states that the memory system used to store information, and *N* is the number of independent physical memories that have been cleared. When a system has acquired the maximal thermodynamic information available in a set of independent binary random variables by recording the outcome of *N* pairs of equally probable events, it can be seen that the above term for minimum energy released becomes *N k*_*B*_*T ln2* joules. Therefore, to find an upper limit on the total number of bits of information that a system can process irreversibly by clearing from its physical memory devices, we will first make two assumptions to describe this hypothetical extreme case. First, we assume that the system in question records new information as efficiently as possible, so that only *k*_*B*_*T ln2* joules of energy is dissipated per bit of information overwritten. Second, we suppose that the information-processing system is noise-free, so that the final state of each of its memories carries zero entropy relative to the system. We can now find an upper limit on information erasure by dividing total energy expenditure Δ*E*_*env*_ by the minimal energy cost of removing one bit (Bormashenko, 2019a). In accordance with Landauer's principle, a system can process at most

(7) $$\begin{array}{l} N\leq \frac{\Delta E_{\text{env}}}{k_{B}T\;ln2} \end{array}$$

bits of thermodynamic information, where *N* is a dimensionless number. Several features of this bound are worth noting. First, this bound holds even in systems using non-binary degrees of freedom to record information (Bormashenko, 2019b). Second, like Landauer's principle itself (Lloyd, 2000), this bound can also be read as stating that a memory device cannot be refreshed if it is not supplied with at least *k*_*B*_*T ln2* joules of free energy per bit intended to be overwritten (Lloyd, 2000). Third, if the information stored by each relevant degree of freedom in a system is calculated to be much smaller than *ln2* bits, then the term on the right side of the inequality above can take arbitrarily large values. When this is the case, the bound becomes less meaningful or only approximate, as the bound itself will not be bounded above. By imposing fundamental limits on energy and information dissipation in physical memories, these equations and inequalities provide convenient ways to study the thermodynamics of information processing in the brain (Collell and Fauquet, 2015; Street, 2016). The next section provides a realistic estimate for the limit above as applied to action potentials in typical neurons in mammalian brains.

## 4. Thermodynamic Information in an Action Potential

According to Landauer's principle, the process of resetting a memory back to its initial state is the key step of irreversible computation that limits information flow through a system (Lent et al., 2018). If we take the view that a neuron is a memory device processing information irreversibly, this step of the cycle of neuronal computation can arguably be identified as the process of re-establishing a resting potential after an action potential has been sent. In other words, if each neuron is a physical memory, the energy used to restore membrane potential is the energy required to reset the neuron to its initial configuration. Further, out of all of the possible configurations that a neuron receiving synaptic inputs may explore in its configuration space, the number of resting and inactive configurations is much larger than the number of actively firing configurations. Therefore, an actively spiking neuron that is found in the relatively improbable state of firing an action potential carries a lower thermodynamic entropy than its initial resting configuration. Taken together, these two observations predict that a neuron returning to its higher-entropy initial state after firing an action potential or spike train must lose information by dissipating energy.

This energy expenditure in neurons is required by the laws of thermodynamics. Just as a Maxwell's demon can store useful energy by recording distinctions about the positions and velocities of particles in a box with a trapdoor separating two compartments, a neuron can store useful energy by recording distinctions about the physical states of ions on either side of its trapdoor-like semipermeable membrane (Aur and Jog, 2007; Sengupta et al., 2013; Davies, 2019). For this reason, in order not to become a perpetual motion machine that could use information to acquire infinite energy, a neuron must expend energy by re-establishing membrane potentials after an action potential has been sent. This process overwrites many previous distinctions carried by the neuron in such a way that forces the neuron to lose information, so it is thermodynamically irreversible. It is in this sense that thermodynamic information is processed during an action potential—any distinctions that a neuron uses to reduce its own thermodynamic entropy over the course of an action potential must ultimately be cleared (Sengupta et al., 2013).

Thus, to find an upper limit for the quantity of thermodynamic information that can be carried by each action potential in a typical mammalian cortical pyramidal cell, we counterintuitively begin by estimating the energy required to restore the membrane potential after an action potential has been sent. Depending on species, cell subtype, and specific morphological characteristics within a given circuit, a typical mammalian cortical pyramidal cell uses between about 10^6^ and 10^10^ molecules of ATP per action potential, with each ATP molecule providing about 10^–19^ joules of useful energy (Attwell and Laughlin, 2001; Nelson, 2004; Pissadaki and Bolam, 2013; Sengupta et al., 2013; Wang et al., 2017). If we assume that ATP is a neuron's primary source of energy for action potential propagation and membrane potential restoration, then the quantity of free energy that is available to process information during the course of an action potential is limited by the number of ATP molecules required by the action potential:

(8) $$\begin{array}{l} \Delta F_{\text{sys}}\leq 10^{10}\;ATP\cdot 10^{-19}\;J/ATP=10^{-9}\;J \end{array}$$

joules of free energy supplied by ATP, per action potential, at most. It should be clarified that this ATP-dependent quantity of energy is not, strictly speaking, the energy required to send an action potential (i.e., the energy cost of opening just enough sodium channels in the axon hillock during passive conduction to initiate full depolarization). Rather, this quantity is an upper-end estimate of the total energy required to prepare the neuron for depolarization by establishing an ion concentration gradient along the entire length of the axon. This line of reasoning is consistent with the idea that spike initiation itself requires relatively little energy, as there is useful energy stored in ion concentration gradients (Zhu et al., 2019). In order not to violate any laws of thermodynamics, a neuron using this quantity of energy to send information with an action potential must ultimately expend at least this quantity of energy, per action potential, over the entire cycle of action potential propagation and membrane potential restoration:

(9) $$\begin{array}{l} \Delta E_{\text{env}}\geq 10^{10}\;ATP\cdot 10^{-19}\;J/ATP=10^{-9}\;J \end{array}$$

joules of energy dissipated by each action potential. Now that we have found a reasonable value for an upper estimate of the numerator in inequality (7), our next step is to find an empirically realistic value for the denominator. For a neuron whose internal temperature is about *T* = 310 K, a realistic value for the denominator in inequality (7) is

(10) $$\begin{array}{l} k_{B}T\;ln2=1.38064852\cdot 10^{-23}\;J/K\cdot 310\;K\;ln2\approx 2.97\cdot 10^{-21}\;J \end{array}$$

joules of energy dissipated per bit of thermodynamic information carried by a maximally informative action potential. If a neuron were to attain this maximum information condition, *k*_*B*_*T ln2* joules of energy would be released into external degrees of freedom by every neuronal subsystem whose change in state meaningfully contributes to the physical implementation of the action potential. We can now divide the maximum energy available for information processing or erasure (9) by the energy cost of overwriting a single bit (10) to find the greatest possible number of bits of thermodynamic information that could be processed irreversibly by a single action potential in a typical neuron at a temperature of 310 K. We find that an action potential can process no more than

(11) $$\begin{array}{l} N\leq \frac{10^{-9}\;J}{2.97\cdot 10^{-21}\;J}=3.37\cdot 10^{11} \end{array}$$

bits of thermodynamic information. If we would prefer to measure these distinctions using Shannon entropy, we can also use the conversion factor 1 Shannon bit = 1/*ln2* thermodynamic bits (natural units, or nats) to find that an action potential can carry at most about 4.86 · 10^11^ bits of Shannon entropy. A few comments are in order regarding some limitations of this result. As one limitation, the number above is not the bound itself, but rather an example of a realistic value for the bound based on the assumption that the neuron it describes requires less than about 10^10^ molecules of ATP per spike in total. As a consequence, although the general form of the bound given by inequality (7) is likely to hold even for atypically energy-demanding neurons, the specific numerical value above may not apply to neurons that use more than 10^10^ molecules of ATP per spike in total. A second limitation is that the specific numerical value for the energy cost of an action potential depends on factors such as ion channel density, degree of myelination, axon length, and axon diameter (Sterling and Laughlin, 2015). These factors are not explicitly incorporated into the bound, so it is very important to accurately estimate the number of ATP molecules required per spike on average when using inequality (7) in order to prevent errors.

As an aside, it is also worth noting the potential existence of a lower bound on the information content of an action potential. While it could be argued that individual ions, electrons, atoms, and other microscopic particles store information simply by carrying distinguishable states, a more modest view is that, at the very least, information is stored in the states of ion channels. If we make a lower-end estimate that a typical mammalian neuron contains 70,000 or more voltage-gated ion channels in total (Buchholtz et al., 2002), and assume that each channel can store up to one bit of information by recording the distinction between whether it is open or closed, then a typical action potential would process at least several tens of thousands of bits of thermodynamic information by altering the states of axonal ion channels. While the idea that a spiking neuron may process such a large quantity of physical information seems to contradict many models of neuronal computation that study more abstract forms of information carried by cells, we should remember that even relatively small systems such as individual atoms and molecules can store large quantities of potential information (Schumacher and Westmoreland, 2010).

In summary, we have arrived at an estimate for the ultimate thermodynamic limit on the quantity of information that can be carried by an action potential in a typical mammalian spiking neuron. By incorporating empirically realistic values of energy dissipation and temperature into the inequality for Landauer's principle, we find that a single action potential in a typical mammalian pyramidal cell can carry no more than about 3.4 · 10^11^ bits of thermodynamic information, or about 4.9 · 10^11^ bits of Shannon entropy. It should be noted that the existence of this upper bound does not necessarily imply that each action potential saturates the bound in terms of the quantity of information that it uses for cell-to-cell signaling or intracellular computation. In much the same way that not every atom, molecule, or subatomic particle must be counted in order to understand the dynamics of neuronal computation at a coarse-grained level, it seems likely that many bits of thermodynamic information involved in the propagation of an action potential can be safely ignored for most practical purposes. Indeed, others have proposed forms of Landauer's principle that explicitly quantify this excess, non-predictive information in neurons (Still et al., 2012). In this regard, the novelty of the present paper is simply to emphasize the magnitude of the total potential information contained in an action potential from a physically pancomputationalist point of view.

## 5. Discussion

While a typical neuronal action potential is commonly treated as carrying no more than a single bit of Shannon entropy, simple thermodynamic arguments suggest that this interpretation may be too oversimplified to be fully consistent with the physical laws of computation. Combining realistic values for neuronal temperature and ATP consumption with the inequality for Landauer's principle shows that a single action potential in a typical mammalian cortical pyramidal cell could hypothetically carry up to approximately 3.4 · 10^11^ bits of thermodynamic information, or approximately 4.9 · 10^11^ bits of Shannon entropy. Clearly, this result challenges the notion that a typical mammalian spiking neuron can be conceptualized as a binary computing element that registers only the information stored in the distinction between whether or not it is firing an action potential at some instant in time.

Yet, while this result contradicts the common neuroscientific assumption each spiking neuron processes information only in the form of abstractly labeled binary states, it arguably finds strong support in the emerging physics of information. If the bit of information in the form of an irreducible distinction is the most fundamental entity in physics (Glattfelder, 2019), it is only natural to hypothesize that neurons process vast quantities of information. The myriad positions, momenta, charges, and other properties of the many interacting constituents of each neuron together hold a large number of bits of potential physical information. Moreover, since a spiking neuron is an open thermodynamic system that decreases its own entropy by dissipating energy, we would expect this information to be processed irreversibly by each action potential. That is, any distinctions carried by the positions, momenta, charges, and other quantities that provide the neuron with useful energy during an action potential must ultimately be lost or erased from the cell and its information-storing subsystems.

Many questions naturally arise from this calculation. For example, if we make the reasonable assumption that spiking neurons are at least somewhat energy-efficient, we are led to conclude that a typical action potential must erase many bits of thermodynamic information. What physical degrees of freedom are being used to store all of these bits? Certainly, a spiking neuron stores many bits of information by recording distinctions about the locations of ions and electrons relative to its axonal membrane. But might the information contained in each action potential also include the degrees of freedom stored in larger physical particles, such as phospholipid molecules or various proteins? While speculative, this possibility would be in line with the proposal that a large quantity of information is processed by the many nuclear spins of phosphorous atoms in neuronal membranes (Smolin, 2020b). As a second question, might this result support the argument (Debanne, 2004) that biologically relevant information is processed by axons? Finally, as a more general question, how can we use other thermodynamic functions and variables to simplify our understanding of neuronal spiking dynamics?

This result also has broader implications for areas of neuroscience beyond the biophysics of cellular computation. From molecular biology to the neuropsychology of consciousness, the concept of neuronal information processing is a central component of a wide range of models and theories in contemporary neuroscience. By showing that a typical action potential can in principle hold a very large quantity of information, this calculation suggests that it would be wise to assume that neurons process information in ways that are more nuanced and sophisticated than we often suppose. How will the assumption that an action potential carries at most one bit of information impede our progress in understanding neuronal information processing? There is no doubt that studying action potentials as simple binary events has led to profound advances in computational neuroscience. But might we be able to build on these advances by studying neurons from a perspective that resonates more closely with the physics of information?

## Author Contributions

The author confirms being the sole contributor of this work and has approved it for publication.

## Conflict of Interest

The author declares that the research was conducted in the absence of any commercial or financial relationships that could be construed as a potential conflict of interest.
